# Ferritinophagy and Ferroptosis in Cerebral Ischemia Reperfusion Injury

**DOI:** 10.1007/s11064-024-04161-5

**Published:** 2024-06-04

**Authors:** Xiaoyue Liu, Canming Xie, Yao Wang, Jing Xiang, Litong Chen, Jia Yuan, Chutao Chen, Haomei Tian

**Affiliations:** School of Acupuncture-moxibustion, Tuina and Rehabilitation, Hunan University of Chinese Medicine, Changsha, 410208 China

**Keywords:** Cerebral ischemia–reperfusion injury, Ferritinophagy, Ferroptosis, NCOA4, Iron metabolism

## Abstract

Cerebral ischemia–reperfusion injury (CIRI) is the second leading cause of death worldwide, posing a huge risk to human life and health. Therefore, investigating the pathogenesis underlying CIRI and developing effective treatments are essential. Ferroptosis is an iron-dependent mode of cell death, which is caused by disorders in iron metabolism and lipid peroxidation. Previous studies demonstrated that ferroptosis is also a form of autophagic cell death, and nuclear receptor coactivator 4(NCOA4) mediated ferritinophagy was found to regulate ferroptosis by interfering with iron metabolism. Ferritinophagy and ferroptosis are important pathogenic mechanisms in CIRI. This review mainly summarizes the link and regulation between ferritinophagy and ferroptosis and further discusses their mechanisms in CIRI. In addition, the potential treatment methods targeting ferritinophagy and ferroptosis for CIRI are presented, providing new ideas for the prevention and treatment of clinical CIRI in the future.

## Introduction

Stroke is the second leading cause of death worldwide, and is characterized by high morbidity, disability, mortality, and recurrence, causing great harm to human life and health [1]. Ischemic stroke accounts for 87% of all stroke cases [2]. The main cause of ischemic stroke is thrombosis in the cerebral blood vessels, which interrupts cerebral blood flow, thus inducing brain cell death, brain tissue necrosis, and neuronal damage [3]. Brain tissue damage can effectively be reduced if thrombolytic treatment is provided within the temporal window; however, reperfusion of blood flow beyond the temporal window may exacerbate the damage by causing cerebral ischemia–reperfusion injury (CIRI) [4]. The pathogenesis of CIRI is complicated, and some studies have pointed out that CIRI may involve multiple modes of cell death, including ferroptosis, autophagy, apoptosis, and necrosis [5]. However, the exact mechanism remains unclear. Therefore, further clarifying the pathogenesis of CIRI and protecting ischemic brain tissues holds significance.

Recent studies have shown that ferroptosis plays an essential role in the development of CIRI. Ferroptosis is a novel mode of iron-dependent cell death. Disorders of iron metabolism leading to intracellular iron overload and overaccumulation of reactive oxygen species (ROS) due to lipid peroxidation are hallmarks of ferroptosis [6]. Ferroptosis has been extensively studied over the past decade, with research focusing on the mechanisms regulating ferroptosis, such as iron metabolism, lipid metabolism, and amino acid metabolism [7]. Furthermore, ferroptosis is an autophagic cell death process, and NCOA4-mediated ferritinophagy serves as an upstream mechanism for inducing ferroptosis by regulating iron metabolism and ROS generation [6]. Ferritinophagy is a selective autophagy process that induces the lysosomal degradation of ferritin, releasing free iron [8]. Under normal physiological conditions, ferritinophagy can maintain the balance of intracellular iron metabolism; however, under the effects of stress factors such as ischemia–reperfusion, ferritinophagy is over-activated to release a large amount of free iron, leading to intracellular iron overload and ferroptosis [9]. Therefore, exploring the mechanisms underlying ferritinophagy and ferroptosis and their role in CIRI may reveal effective treatments for CIRI.

Clinical treatments for CIRI are currently limited, highlighting the importance of developing effective therapeutic approaches. Ferritinophagy and ferroptosis represent important pathogenic mechanisms in the development of CIRI; hence, interventions targeting ferroptosis are being studied, including Western drugs and traditional Chinese medicines. In contrast, fewer studies have investigated ferritinophagy as a potential treatment target against CIRI, but ferritinophagy remains an important link to ferroptosis. Acupuncture, a traditional treatment in Chinese medicine, can alleviate CIRI through a variety of mechanisms, including anti-inflammatory response, oxidative stress, autophagy, apoptosis, ferroptosis, and other mechanisms [10, 11]. The previous research of our experimental group also confirmed that acupuncture could inhibit ferroptosis in rat neuronal cells after CIRI, and could alleviate CIRI by regulating related signaling pathways to activate or inhibit autophagy [12]. Nevertheless, whether acupuncture can modulate autophagy to inhibit ferroptosis has not been clarified. Considering the pathophysiological association between autophagy and ferroptosis, acupuncture is hypothesized to inhibit ferroptosis after CIRI by modulating NCOA4-mediated ferritinophagy, which might provide an effective target for the treatment of CIRI.

This review mainly summarizes the occurrence and regulatory mechanisms of ferritinophagy and ferroptosis, illustrating the relationship between the two, and further discussing the mechanisms of ferritinophagy and ferroptosis involved in CIRI. Moreover, the potential treatment methods targeting ferritinophagy and ferroptosis for CIRI are described, providing new ideas for the prevention and treatment of clinical CIRI in the future.

## Mechanism and Regulation of Ferritinophagy and Ferroptosis

**Fig. 1:**
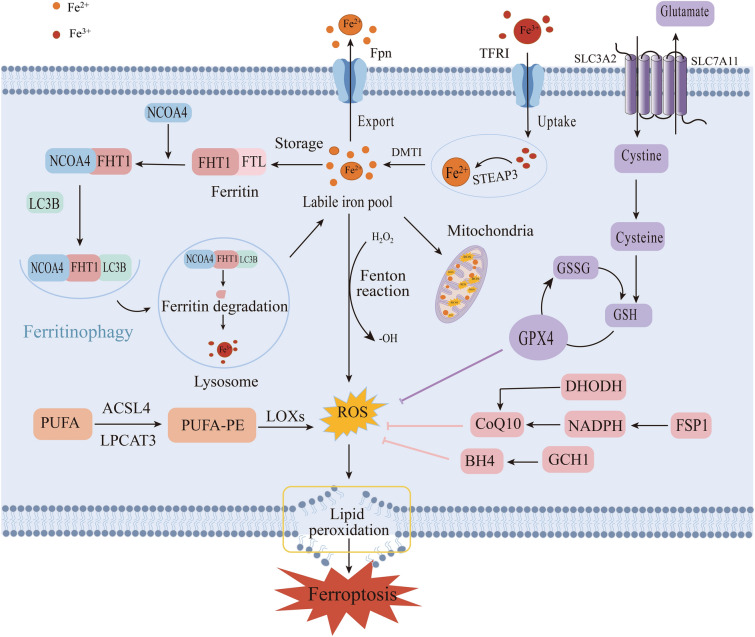
Process and mechanisms of ferritinophagy and ferroptosis. The mechanism of ferroptosis involves four aspects, iron metabolism, lipid metabolism, the antioxidant system centered on GPX4, and other antioxidant factors. NCOA4-mediated ferritinophagy is a selective autophagic modality targeting ferritin degradation, and the LC3B-mediated autophagic lysosomal degradation pathway is the classical pathway, which primarily regulates iron metabolism targeting ferroptosis from upstream mechanisms

Ferroptosis is a mode of programmed cell death induced by iron-dependent oxidative damage and is mainly characterized by iron overload, excessive accumulation of ROS, and lipid peroxidation [6]. Ferritinophagy, a selective autophagy modality involving ferritin degradation, can regulate iron metabolism to prevent lipid peroxidation resulting from iron overload, thereby inhibiting ferroptosis. Ferritinophagy and ferroptosis are closely related, and ferritinophagy regulates ferroptosis (Fig. 1).

## Mechanism and Regulation of Ferroptosis

### Iron Metabolism

Iron is an essential micronutrient for the maintenance of life and exists in the human body mainly in the form of divalent and trivalent iron [13, 14]. In the acidic environment of endocytosed vesicles, the Fe^3+^ separates from transferrin and is reduced to Fe^2+^ by six-transmembrane epithelial antigens of prostate 3, which is released into the labile iron pool in the cytoplasm through the divalent metal transporter 1 (DMT1) [15]. While the Fe^2+^ in the cytoplasm can be oxidized to Fe^3+^ by membrane iron transporter 1 and conveyed to the extracellular space, the excess iron is stored in ferritin, which maintains the iron metabolic circulation and achieves intracellular iron homeostasis [16]. However, the disruption of iron metabolism leads to the production of toxic hydroxyl radicals, and highly reactive ROS are generated through the Fenton reaction, inducing lipid peroxidation and ferroptosis [17]. Iron chelators are widely used in clinical practice to inhibit iron overload, such as desferrioxamine and cyclopiazide amine, which inhibit ferroptosis by converting free iron into stable complexes and reducing lipid peroxidation [6, 16]. In conclusion, iron is an essential element for ferroptosis, and disorders of iron metabolism are crucial in causing intracellular iron overload and inducing ferroptosis.

### Lipid Metabolism

Iron ion-dependent membrane lipid peroxidation is a pivotal mechanism to induce ferroptosis [18]. Studies have confirmed that lipids containing polyunsaturated fatty acids (PUFAs) located in the cell membrane are drivers of ferroptosis; in addition, highly oxidizing ROS react with cell membrane lipids and are involved in lipid peroxidation [7]. Acetyl coenzyme A (CoA) synthetase long-chain family member 4 (ACSL4) and lysophosphatidylcholine-acyltransferase 3 (LPCAT3) are critical enzymes for the regulation of fatty acids, which activate PUFAs and promote their binding to membrane lipids, increasing cellular peroxidative damage and contributing to ferroptosis [19]. Inhibition of the activities of ACSL4 and LPCAT3 can inhibit ferroptosis accordingly [20]. Furthermore, lipoxygenase (LOX) mediates ferroptosis by catalyzing lipid peroxidation [19]. Existing studies have reported that lipid ROS inhibitors such as Vitamin E, Ferrostatin-1, and Liproxstatin-1 effectively cleared excess ROS in the cytoplasm and inhibited ferroptosis [21]. Moreover, studies found that Zileuton, a 5-LOX inhibitor, could prevent glutamate oxidative toxicity, inhibit lipid ROS and ferroptosis, and protect mouse hippocampal HT22 neuronal cells [22].

### GPX4-centered Antioxidant System

Glutathione peroxidase 4 (GPX4) is a specialized antioxidant enzyme that is regulated by system Xc^−^ [23]. System Xc^−^ is a reverse transporter system that mediates the entry of extracellular cystine into the cell and controls the excretion of intracellular glutamate, which is regulated by two proteins, namely solute carrier family 7 member 11 (SLC7A11) and solute carrier family 3 member 2 [24]. Glutathione (GSH) is an essential co-factor of GPX4 that directly affects the activity of GPX4, which is responsible for catalyzing the reduction of toxic lipid peroxides to non-toxic fatty alcohols, thereby negatively regulating ferroptosis. Of the three amino acids found in GSH, cysteine is usually the least abundant and is considered to be the rate-limiting factor in the synthesis of GSH [25]. In conclusion, the System Xc^−^/GSH/GPX4 signaling pathway axis is generally regarded as a crucial axis for the regulation of ferroptosis. Potential therapeutic compounds have been developed around three inducer targets, including systemic Xc^−^inhibitors, GPX4 inhibitors, and GSH-depleting agents [26]. These inducers exert pro-ferroptosis effects by preventing System Xc^−^ transport of cystine and glutamate through related mechanisms [27, 28], or by inhibiting the expression of the antioxidant enzyme GPX4 [29–31].

### GPX4-independent Related Regulatory Mechanisms

In addition to the above GPX4-centered regulatory system, three ferroptosis-independent regulatory pathways have been identified in recent years, namely, ferroptosis suppressor protein 1 (FSP1)/Coenzyme Q10 (CoQ10), dihydrolactate dehydrogenase (DHODH), and GTP cyclohydrolase 1 (GCH1)/tetrahydrobiopterin (BH4) [7]. CoQ10 is a lipid-soluble antioxidant; FSP1 modified by cardamonylation was found to utilize NADPH to reduce CoQ10 on the plasma membrane to ubiquinol (reduced CoQ10) to trap free radicals, block lipid peroxidation, and inhibit cell ferroptosis [32, 33]. Hence, the FSP1-NADPH-CoQ10 pathway plays a critical role in inhibiting ferroptosis. Moreover, DHODH is a ferroptosis inhibitor located in mitochondria that can reduce CoQ10; high expression of DHODH was reported to inhibit ferroptosis [34]. Furthermore, in CRISPR screens, GCH1 was found to generate the endogenous metabolite BH4 to inhibit ferroptosis [35]. The GCH1-BH4-phospholipid axis may represent a protective mechanism independent of GSH/GPX4 [36]. In-depth research on the ferroptosis regulation mechanism can provide an effective reference for clinical targeting of ferroptosis to treat related diseases.

## Mechanism and Regulation of Ferritinophagy

The term “Ferritinophagy” was coined in 2014, and Mancias et al. used a combination of autophagic vesicle isolation and quantitative proteomics to identify NCOA4 as a pivotal receptor protein mediating ferritinophagy [8]. Subsequently, NCOA4 was found to interact with the ferritin complex and be involved in lysosomally targeted ferritin degradation [37]. Ferritin is an iron storage protein, comprised of ferritin heavy chain 1 (FTH1) and ferritin light (FTL), which can maintain cellular iron homeostasis by storing and releasing iron. Studies have demonstrated that FTH1 is more likely to bind and release iron compared to FTL [37, 38]. When cellular iron levels become excessively low, ferritin can be activated to undergo autophagy. NCOA4 interacts with the conservative surface arginine (R23) on FTH1 to promote the formation of autophagic vesicles after interacting with the autophagy-related gene ATG8-like protein microtubule-associated protein 1 light chain 3B (LC3B). Ferritin is then carried to the lysosome for degradation, completing ferritinophagy [39].

The level of ferritinophagy is determined by NCOA4, which in turn is regulated by a variety of factors, including iron content, autophagy, lysosomes, and hypoxia. The E3 ubiquitin protein ligase 2 (HERC2)-mediated NCOA4 ubiquitination pathway plays an essential role in the regulation of ferritinophagy. Moreover, the C-terminal structural domain of the NCOA4 protein has binding sites for FTH1 and HERC2 [40]. Mancias et al. revealed that iron ions can negatively regulate NCOA4 levels in a variety of cell lines. High intracellular iron ion concentrations trigger the interaction between the HECT and RLD structural domains of HERC2, mediating the ubiquitination of NCOA4 and promoting NCOA4 degradation via the ubiquitin–proteasome system, which reduces ferritinophagy flux. Conversely, low intracellular iron ion concentrations inhibit the binding of NCOA4 and HERC2, thereby increasing ferritinophagy levels [41]. The HERC2-FBXL5-IPR2 axis participates in modulating NCOA4-mediated ferritinophagy and iron metabolism [42]. Notably, the mutual combination of NCOA4 and FTH1 is a necessary process for ferritinophagy. Ferritinophagy can be directly inhibited by NCOA4 knock out, knock down, or blocking the binding of NCOA4 to FTH1. In addition, compound 9a and Yes-associated protein 1 (YAP1) were shown to target and block NCOA4-FTH1 binding and inhibit ferritinophagy [39, 43].

**Fig. 2:**
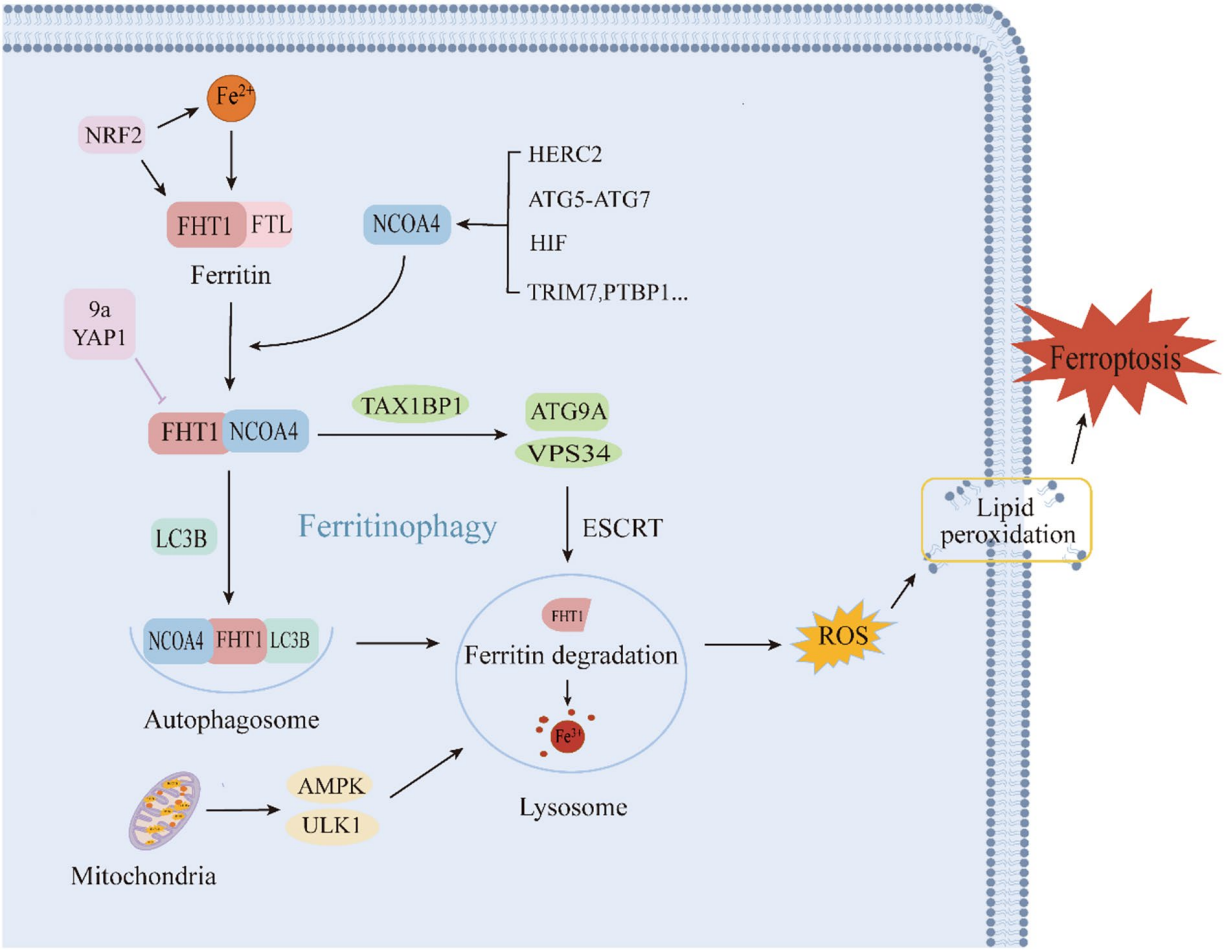
Mechanism and regulation of ferritinophagy and the relationship between ferritinophagy and ferroptosis. Mutual binding of NCOA4 and ferritin delivered to the lysosome for degradation is a key process of ferritinophagy, which is regulated by the LC3B-mediated autophagy pathway as well as the ESCRT-mediated non-autophagic pathway. NCOA4 is a critical protein for ferritinophagy, and NCOA4 is regulated by a variety of factors including iron content, autophagy, lysosomes, and hypoxia

Ferritin is generally degraded by two mechanisms, namely lysosomal and proteasomal processes. NCOA4-FTH1 translocation to lysosomal degradation is a crucial mechanism of ferritinophagy [44]. In addition to the classical LC3B-dependent autophagy pathway, NCOA4-Ferritin transfer to lysosomal degradation also includes the ESCRT-mediated non-autophagic lysosome-targeted pathway, which requires the joint involvement of TAX1BP1, VPS34, the ATG9A complex, and so on [45]. Moreover, hypoxia has been shown to modulate ferritinophagy, while hypoxia-inducible factor (HIF) can regulate NCOA4, and increased HIF1α or HIF2α activity in cells can raise NCOA4 mRNA levels [46]. In addition to the above regulatory pathways, many NCOA4-related signaling molecular pathways are involved in regulating ferritinophagy. For example, modulation of the TRIM7-k48-NCOA4 pathway reduces NCOA4-mediated ferritinophagy in human glioblastoma cells [47]. In addition, polypyrimidine pathway binding protein 1 (PTBP1) has been shown to mediate ferroptosis in hepatocellular carcinoma cells by regulating the translation of NCOA4, and the PTBP1-NCOA4 axis may be an important pathway for ferritinophagy-mediated ferroptosis [48]. Mechanism and regulation of ferritinophagy and the relationship between ferritinophagy and ferroptosis in Fig. 2.

## Ferritinophagy and Ferroptosis

Autophagy is an intracellular defense and strain regulation mechanism that is involved in the degradation of damaged proteins and organelles through the lysosomal pathway [49]. Ferroptosis is an autophagic cell death process [50], and several selective autophagic modalities, such as NCOA4-mediated ferritinophagy, RAB7A-mediated lipophagy, ARNTL-mediated coprophagy, PINK1-mediated mitochondrial autophagy, and HSP90-mediated CMA, have been found to promote ferroptosis [51]. Among them, NCOA4-mediated ferritinophagy is a crucial upstream mechanism for inducing ferroptosis by regulating cellular iron metabolism and ROS. Knockout or knockdown of NCOA4 was found to limit ferritin degradation and consequently inhibit ferroptosis [40]. Gao et al. reported that the ATG5-ATG7-NCOA4 autophagy pathway may be a key pathway regulating ferritinophagy-mediated ferroptosis [40, 50]. Surprisingly, ROS originating from mitochondria triggered ferritinophagy by activating AMP-activated protein kinase (AMPK) and unc-51-like autophagy-activated kinase 1 axes, leading to a further increase in intracellular free iron and ROS [52]. This axis represents an alternative pathway for ferritinophagy-induced ferroptosis. In conclusion, ferritinophagy is a potential upstream target of ferroptosis, and NCOA4-mediated ferritinophagy may be an important link in inducing ferroptosis.

Recent studies have revealed that nuclear factor red lineage 2-related factor 2 (NRF2) may be a potential target for the regulation of ferritinophagy and ferroptosis. Nrf2 is a basic leucine zipper transcription factor that promotes the transcription of related genes and regulates oxidative stress [53]. NRF2 is intimately associated with iron metabolism, which can modulate the expression of iron metabolism-related proteins such as TfR1, ferritin, and Fpn, thus affecting ferritinophagy and ferroptosis [54]. Furthermore, NRF2 not only promotes ferritin expression and reduces unstable iron levels [55], but also regulates gene transcription of GSH and GPX4 and inhibits ferroptosis [56, 57]. Ma et al. revealed that NRF2 inhibited NCOA4-mediated ferritinophagy to reduce cartilage endplate chondrocyte ferroptosis and decrease intervertebral disc degeneration disease [58]. Meanwhile, Liu et al. also confirmed the role of NRF2 in ferritinophagy and ferroptosis in a related study exploring macrophage inflammation [59]. Additionally, cerebral ischemia was found to produce large amounts of ROS that activate NRF2 and stimulate the transcription of the antioxidant genes heme oxygenase-1 (HO-1), and others [60]. In a middle cerebral artery occlusion/reperfusion (MCAO/R) model, NRF2 was found to be upregulated at 3 h after ischemia and peaked at 24 h [61]. Modulation of NRF2-associated signaling pathways attenuates ferritinophagy and ferroptosis after cerebral ischemia/reperfusion, with the Keap1-NRF2-HO-1 signaling pathway being a crucial pathway to combat oxidative stress and regulate ferroptosis after CIRI [62].Therefore, NRF2 might be a potential target for regulating ferritinophagy and ferroptosis.

## Ferritinophagy and Ferroptosis Participate in the Development and Regulatory Mechanisms of CIRI

### Ferroptosis and CIRI

Disturbances in iron metabolism result in cellular iron overload and can cause membrane lipid peroxidation, ultimately leading to ferroptosis. This process represents a critical mechanism of neuronal injury in cerebral ischemia–reperfusion. Divalent iron levels exceeding 0.2 mmol/l have been shown to cause brain tissue damage [63]. According to previous studies, cerebral ischemia–reperfusion (CIR) can increase ferroportin by activating the JAK/STAT3 pathway or upregulating the expression of HIF-1α, leading to iron overload in the brain [64]. Moreover, iron contributes to the development of CIRI primarily via the following pathways. Firstly, iron can directly catalyze the generation of free radicals, especially through the Fenton reaction, which is converted into more reactive hydroxyl radicals; free radicals attack the unsaturated fat chains on the cell membranes, resulting in lipid peroxidation. This process not only occurs immediately after cerebral ischemia/reperfusion but also persists for a long period after the ischemia/reperfusion and promotes neuronal death [65, 66]. On the other hand, iron itself is toxic; it can damage lysosomal and mitochondrial membranes, and the reduction of intracellular Fe^3+^ to Fe^2+^ can directly cause DNA denaturation [67].

The role of iron-ion-dependent cellular ferroptosis in CIRI has received widespread attention in recent years, with a growing number of studies suggesting that ferroptosis plays a vital role in CIRI [68]. For instance, an experiment employing a rat MCAO/R model showed that rats developed serious cerebral injury and neurological defects after reperfusion, displaying typical molecular features of ferroptosis, including iron overload, GSH disorders, and increased lipid peroxidation, among others [69]. Similarly, a significant occurrence of ferroptosis was detected in the ischemic side of the brain tissue in mice, and inhibition of ferroptosis with the ferroptosis inhibitors Liproxstatin-1 or Ferrostatin-1 markedly reduced the size of cerebral infarcts and lowered the CIRI [70].

In practice, iron chelators like deferoxamine are extensively used, and although several animal studies have revealed the ability of iron chelators to attenuate iron overload in CIRI, the clinical efficacy of these medications remains elusive and requires further detailed research [71]. The role of ferritin in CIRI cannot be overlooked. Ferritin over-expression can alleviate CIRI, whereas reduced ferritin levels increase p53 and SLC7A11-mediated ferroptosis and promote neuronal injury after cerebral ischemia [72].Cellular ferroptosis following cerebral ischemia and reperfusion is also regulated by multiple molecular mechanisms and signaling pathways. Research showed that inhibition of NLRP3 inflammatory vesicles may inhibit ferroptosis and inflammation through the Keap1-Nrf2 pathway, thereby alleviating CIRI [73]. Elabela (ELA) is a new endogenous ligand for the Apelin receptor (APJ), and the Elabela-APJ axis has been shown to alleviate CIRI by inhibiting neuronal ferroptosis. Therefore, the ELA-32 peptide might be an effective treatment pathway for cerebral ischemia [74]. Fan et al. then reported that GATA-binding protein 6 (GATA6) was associated with CIRI using bioinformatics prediction, and GATA6 may inhibit neuronal autophagy and ferroptosis through the miR-193b/ATG7 axis, thereby attenuating brain I/R injury [75]. In addition, the SSAT1/ALOX15 axis is associated with neuronal ferroptosis after CIRI [76].

In conclusion, ferroptosis is an important mechanism in the development of CIRI, whereas inhibition of ferroptosis decreases the injury accordingly. As a novel cell death modality distinct from conventional autophagy, apoptosis, and necrosis, ferroptosis is a potential target for the treatment of CIRI.

### Ferritinophagy and CIRI

Ferritinophagy mainly regulates iron metabolism and controls ferroptosis from upstream mechanisms. NCOA4-mediated ferritinophagy releases large amounts of free iron by targeting ferritin, leading to disturbed iron metabolism and promoting ferroptosis after CIRI. Brain tissue ischemia and hypoxic necrosis were detected following CIRI, with elevated NCOA4 expression and enhanced levels of ferritinophagy. In contrast, the use of ferritinophagy inhibitors or gene silencing of NCOA4, a key protein in ferritinophagy, resulted in a reduction in the infarct volume of the brain tissue, an increase in neurological function scores, and a reduction in CIRI [77]. A previous study revealed that ferritinophagy-induced degradation of ferritin was the highest at 6 h of reperfusion; thus, targeting ferritinophagy in this time window may be an effective treatment approach for CIRI [78]. Thus, ferritinophagy is considered one of the critical mechanisms involved in CIRI.

A few studies have investigated the regulatory mechanisms of ferritinophagy following CIRI, and current studies mainly focus on ubiquitin-specific peptidase 14 (USP14) and the cyclic guanylate adenylate synthetase (cGAS)-stimulator of interferon genes (STING) signaling pathway. USP14, an initiator of ferritinophagy induction, upregulates NCOA4 levels in neurons [51]. In an in vivo mouse MCAO/R model and an in vitro cytosolic oxygen–glucose deprivation/reperfusion model, USP14 was found to significantly upregulate NCOA4 levels. Moreover, the use of IU1, an inhibitor of USP14, significantly reduced NCOA4 levels in neurons and inhibited ferritinophagy, thus reducing brain tissue damage after CIR [77]. The cGAS-STING signaling pathway is a classical immune signaling pathway involved in the early pathological process of CIRI [79]. Li et al. reported that early cerebral ischemia–reperfusion activates NCOA4-mediated ferritinophagy, induces oxidative stress, and exacerbates brain injury. Furthermore, a cGAS inhibitor was administered to NCOA4-overexpressing mice, demonstrating that inhibition of the cGAS-STING pathway reduced ferritinophagy, decreased oxidative stress, autophagy, and apoptosis, and ameliorated CIRI [78].

**Fig. 3:**
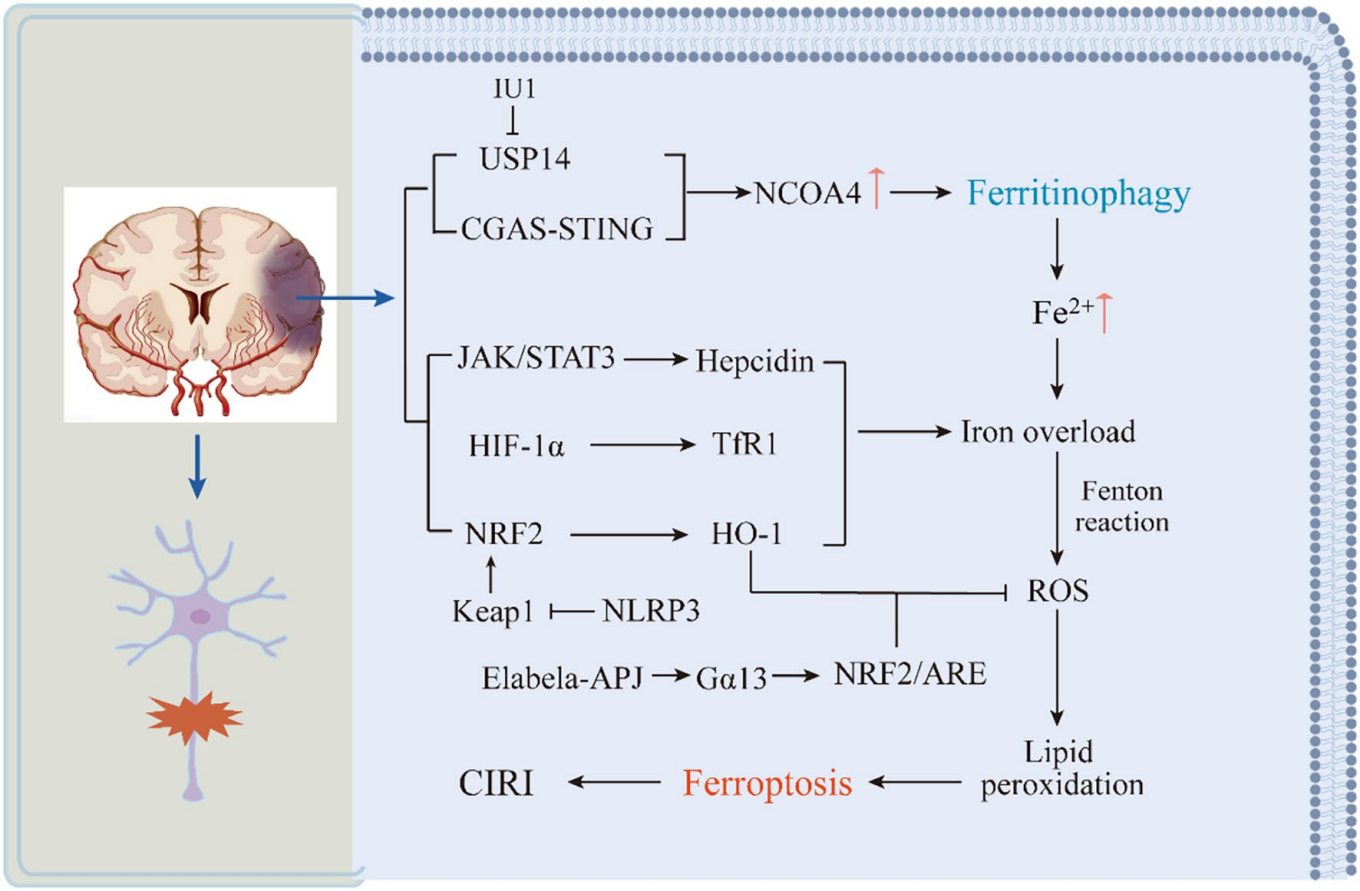
Mechanisms of ferritinophagy and ferroptosis involved in CIRI. Cerebral ischemia–reperfusion induces NCOA4-mediated ferritinophagy and contributes to iron overload through related signaling pathways. Ferritinophagy is a critical mechanism that contributes to iron overload and lipid peroxidation-induced ferroptosis in neuronal cells, which leads to CIRI. In addition, NRF2 is an important regulator of antioxidants

The above suggests that USP14 inhibitors and cGAS inhibitors play an essential role in ferritinophagy for CIRI treatment. However, only a few interventions directly targeting ferritinophagy in CIRI have been developed, and the time-point studies are limited to the first 6 h following reperfusion. The pathophysiological response of ferritinophagy at later time points remains unknown and requires further study. The specific mechanisms of ferritinophagy and ferroptosis involved in CIRI are described in Fig. 3.

## Potential Treatments Targeting Ferritinophagy and Ferroptosis for CIRI

### Targeting Ferroptosis for CIRI

Interventions aiming to target ferroptosis for CIRI have been researched in recent years. Significant progress has been made in the research of Western and traditional Chinese medicines and some special compounds, as well as in acupuncture. Based on the analysis of effective drugs researched currently, butylphthalide (NBP) inhibits ferroptosis after CIRI and protects neurons via the SCL7A11-GPX4 axis [80]. Dexmedetomidine (DEX), an anesthetic drug, has also been shown to have this effect [81]. Furthermore, sodium selenite preconditioning can ameliorate hyperglycemia-mediated CIRI by activating the Hippo pathway to regulate ferroptosis [82]. In contrast, propofol suppresses OGD/R-induced neuronal ferroptosis by inhibiting the HIF-1α/YTHDF1/BECN1 axis [83]. In traditional Chinese medicine, Paeoniae Rubra, the root of Paeonia lactiflora, was found to activate autophagy and inhibit ferroptosis via the PI3K/Akt signaling pathway to alleviate CIRI [84]. Astragalus propinquus (RAP), which is the monarch drug of Buyang Huanwu decoction, can treat stroke and cerebral ischemia, etc. RAP was found to modulate transmembrane iron transport and ferroptosis, improving CIRI [85].

Some Chinese medicinal agents have been approved by the Chinese National Drug Administration for clinical use in the treatment of stroke, including the Xingnaojing injection [86] and Compound Tongluo Decoction (CTLD), which can regulate ferroptosis-related proteins (GPX4, HO-1, and so on) [87] or the related signaling pathway (Nrf2/ARE/SLC7A11) [88] to inhibit ferroptosis and improve the CIRI. The Naotaifang formula attenuates OGD/R-induced inflammation and ferroptosis by mediating the BMP6/SMADs signaling pathway to modulate microglia M1/M2 polarization [89]. Moreover, Danlou tablet not only has pharmacological effects such as anti-inflammatory and anti-oxidative stress but also inhibits ferroptosis after CIRI and reduces blood–brain barrier damage [90].In some herbal extracts, Kang et al. found that the combination of Astragaloside IV (AST IV) and Panax notoginseng saponins (PNS) activated Nrf2 to inhibit ferroptosis and attenuate brain tissue injury after CIRI [91, 92]. Neutral polysaccharides from Gastrodia elata (NPGE), an active ingredient of Gastrodia elata, have also been shown to exert the same effect via the NRF2/HO-1 signaling pathway [93]. Similarly, baicalein and puerarin, the main active components of the traditional Chinese medicine Baikal Skullcap and Pueraria lobata root, can attenuate CIRI by regulating autophagy or ferroptosis-associated proteins [94, 95].

In addition, many specific compounds have been proven to target ferroptosis for CIRI. Procyanidin (PC) is not only a polyphenolic antioxidant but also a potent metal chelator; PC has been shown to inhibit ferroptosis and attenuate CIRI by activating the Nrf2/HO-1 pathway [96]. Furthermore, Rhein exerted the same effect through the NRF2-SLC7A11-GPX4 axis [97]. Similarly, some flavonoids such as soybean isoflavones (SI) [98], vitexin [99], galangin [100], and kaempferol (KF) [101] can also inhibit ferroptosis via corresponding mechanisms and pathways, providing new perspectives for the treatment of CIRI. Meanwhile, pretreatment with some selenium compounds, such as methylselenocysteine or selenocysteine, can prevent ferroptosis after CIRI [102]. Multiple studies have reported that compounds such as carvacrol (CAR) [68], resveratrol [103], β-caryophyllene [104], and Loureirin C [105] can also target and intervene in ferroptosis to attenuate brain tissue damage after reperfusion. In addition, other therapeutic agents may provide potentially effective measures to intervene in ferroptosis after CIRI. The thrombin-ACSL4 axis is a critical target for the inhibition of ferroptosis in the treatment of ischemic stroke, and antithrombin therapeutics may be beneficial in inhibiting ferroptosis after CIRI [106]. Folic acid (FA) is an important nutrient in the human body, and FA supplementation reduces the risk of stroke and may also inhibit ferroptosis after CIRI by regulating glutamate carboxypeptidase transcriptional adaptive response [107]. Furthermore, previous studies have reported that fat-soluble solvent-cottonseed oil (CSO) [108], hyperbaric oxygen [109], and other methods can attenuate CIRI by specific mechanisms related to ferroptosis.

Acupuncture has been recognized by the World Health Organization (WHO) as an effective treatment for CIRI, but its exact mechanism remains elusive. In recent years, studies have revealed that electroacupuncture (EA) can be used to treat CIRI, which inhibits ROS production and ferroptosis [110]. EA pretreatment also demonstrated similar effects [111]. In addition, Zhang et al. found that moxibustion was able to reduce iron deposition in rat hippocampal tissues by regulating iron metabolism, lowering the level of lipid peroxidation, and inhibiting ferroptosis after CIRI, thereby exerting a neuroprotective effect [112]. Therefore, acupuncture represents an effective means of clinically targeting ferroptosis in the treatment of CIRI. In conclusion, ferroptosis is a target for the treatment of CIRI, and multiple studies have researched methods to target ferroptosis for the treatment of CIRI, aiming to develop accurate, efficient, and side-effect-free therapeutic methods for the clinical treatment of CIRI. Potential therapies for targeting ferroptosis for the treatment of CIRI are summarized in Table 1.

**Table 1:** Potential therapies to target ferroptosis for the treatment of CIRI

Potential treatments	Representative drugs	Related mechanisms	References
Western medicine	Butylphthalide, Dexmedetomidine	By increasing expression of SCL7A11 and GPX4 to inhibit ferroptosis	[80, 81]
	sodium selenite	By activating the Hippo pathway to resist ferroptosis after CIRI	[82]
	Propofol	By inhibiting the HIF-1α/YTHDF1/BECN1 axis to suppress OGD/R-induced neuronal ferroptosis	[83]
Traditional Chinese medicine	Paeoniae Rubra	By activating PI3K/Akt signaling pathway to induce autophagy and inhibit ferroptosis	[84]
	Astragalus propinquus	By regulating transmembrane iron transport and ferroptosis to improve CIRI	[85]
Proprietary Chinese medicine	Xingnaojing	By modulating ferroptosis-related proteins such as GPX4, HO-1, FPN, and DMT1 to inhibit ferroptosis	[86]
	CTLD	By regulating the Nrf2/ARE/SLC7A11 pathway to inhibit ferroptosis after CIRI	[88]
	Naotaifang	By mediating the BMP6/SMADs signaling pathway to reduce OGD/R-induced inflammation and ferroptosis	[89]
	Danlou tablet	By reducing oxidative stress and COX2 protein levels, increasing SLC7A11 and GPX4 proteins to inhibit ferroptosis	[90]
Traditional Chinese medicine extract	AST IV and PNS	By activating the NRF2 pathway to inhibit ferroptosis and alleviate brain damage caused by CIR	[92]
	NPGE	By activating NRF2/HO-1 signaling pathway to inhibit ferroptosis after CIRI	[93]
	Baicalein	By regulating GPX4/ACSL4/ACSL3 axis to relieve iron death after CIRI	[94]
	Puerarin	By regulating autophagy and ferroptosis-related proteins to attenuate CIRI	[95]
Compounds	Procyanidins, Rhein, SI, Vitexin, Galangin, KF, carvacrol, et al	By modulating NRF2-associated signaling pathways to attenuate oxidative damage or improve cerebral blood flow and inhibit ferroptosis	[68, 96–105]
Others	Thrombin, FA, CSO, HBO	By regulating the expression of ferroptosis-related proteins to attenuate CIRI	[106–109]
Acupuncture therapy	Electroacupuncture	By decreasing ACSL4 and TfR1 expression and promoting GPX4 levels to inhibit ROS production and ferroptosis	[110]
	Electroacupuncture pretreatment	By modulating iron metabolism or increasing GSH/GPX4 expression to reduce oxidative stress after CIRI	[111]
	Moxibustion	By reducing levels of lipid peroxides, malondialchehyche, ACSL4, hepcidin to inhibit ferroptosis	[112]

*SLC7A11* solute carrier family 7 member 11, *GPX4* glutathione peroxidase 4, *CIRI* Cerebral ischemia–reperfusion injury, *OGD/R* oxygen–glucose deprivation/reperfusion, *HO-1* heme oxygenase-1, *DMT1* Divalent metal transporter 1, *FPN* Membrane iron transporter, *CTLD* Compound Tongluo Decoction, *NRF2* nuclear factor E2 related-factor 2, *AST IV* astragaloside IV, *PNS* Panax notoginseng saponins, *NPGE* Neutral polysaccharide from Gastrodia elata, *ACSL4* long chain fatty-acid CoA ligase 4, *SI* Soybean isoflavones, *KF* Kaempferol, *FA* Folic acid, *CSO* Cottonseed oil, *HBO* Hyperbaric oxygen, *TfR1* transferrin receptor 1, *ROS* reactive oxygen species, *GSH* glutathione

## Therapeutic Approaches to Target Ferritinophagy

Ferritinophagy is an upstream regulatory mechanism of ferroptosis and a direct causative factor for a variety of diseases. Targeting ferritinophagy for the treatment of related diseases has been widely discussed. NCOA4 is a key protein in ferritinophagy, and NCOA4 plays an essential role in the regulation of DNA replication, cell proliferation, and tissue regeneration [113]. In Western medicine research, the anti-malarial drug artesunate (ART) was found to degrade ferritin through autophagic lysosomal function, inducing cell death. In contrast, the knockdown of NCOA4 expression decreased ART-induced cell death [114]. DEX effectively inhibited methotrexate (MTX)-induced neurotoxicity and inflammation in the hippocampal HT22 cell line via NCOA4-mediated ferritinophagy, and alleviated chemotherapy-induced cognitive impairment (CICI) [115]. Cyclosporin A (CsA) mediates HuR translocation to inhibit ferritinophagy to attenuate inflammation and apoptosis in neuronal cells, thereby attenuating cognitive impairment in CICI mice [116]. Moreover, NBP was mentioned as a drug targeting ferritinophagy for the treatment of CIRI. Ye et al. explored its mechanism and found that Dl-3-n-butylphthalide NBP could regulate the expression of Nrf2 in the nucleus, inhibit ferritinophagy, and treat neurodegenerative disorders such as Parkinson’s disease [117], providing a new therapeutic strategy.

Curcumol is a component extracted from the traditional Chinese medicine turmeric. Studies have demonstrated that curcumol can target YAP/NCOA4 to regulate ferritinophagy against hepatocyte senescence for the treatment of non-alcoholic fatty liver disease (NAFLD) [118]. Schisandrin B (Sch B), an active ingredient from Schisandra chinensis, exhibits antitumor, antioxidant, anti-inflammatory, and hepatoprotective properties, which can promote the senescence of activated hepatic stellate cells, ameliorating liver fibrosis by inducing NCOA4-mediated ferritinophagy and iron overload [119]. Likewise, Oroxylin A, an active constituent of Scutellaria baicalensis, can regulate ferritinophagy and inhibit hepatic fibrosis via the cGAS-STING pathway [120]. In addition, some potent compounds, such as Corilagin, have anti-inflammatory and antioxidant effects [121, 122], Wang et al. found that Corilagin could inhibit NCOA4-mediated ferritinophagy, regulate iron homeostasis, inhibit ferroptosis, and attenuate intestinal ischemia/reperfusion injury (IIRI) in mice [123]. A coumarin derivative extracted from a variety of plants, Esculetin (6,7-dihydroxycoumarin), was shown to induce ferritinophagy in hepatocellular carcinoma cells through the NCOA4/LC3II/FTH1 pathway, promoting ferroptosis and exerting anticancer effects [124]. In conclusion, the above drugs and components can modulate ferritinophagy for the treatment of diseases; still, studies focusing on ferritinophagy-modulating drugs for the treatment of CIRI are lacking.

Although no effective drugs have been developed to directly target ferritinophagy for the treatment of CIRI, preliminary studies have been conducted. Ginkgolide B (GB), derived from the leaves of the Ginkgo biloba tree in China, which is a biologically active terpene lactone, can be used as an herbal supplement to promote vascular health and cognitive function. Importantly, a recent study revealed that GB can inhibit ferritinophagy and alleviate brain I/R injury by disrupting the NCOA4-FTH1 interaction [125]. This study suggests that GB may be a therapeutic option for clinically targeting ferritinophagy to treat CIRI. In addition, multiple studies have demonstrated that acupuncture can treat and alleviate CIRI by targeting ferroptosis. Since ferritinophagy is a key upstream mechanism for inducing ferroptosis, acupuncture may be an effective means of targeting ferritinophagy to treat CIRI, but this hypothesis requires further research. Potential therapies for targeting ferritinophagy for related diseases are shown in Table 2.

**Table 2:** Potential therapies to target ferritinophagy for related diseases

Potential treatments	Related mechanisms	Diseases	References
ART	By autophagic lysosomal function to degrade ferritin and induce cell death	Malaria, cancer	[114]
DEX	By decreasing FTH1 expression and increasing NCOA4 content to inhibit ferritinophagy	CICI	[115]
CsA	By mediating HuR translocation to inhibit ferritinophagy and attenuate inflammation and apoptosis in neuronal cells	CICI	[116]
NBP	By regulating Nrf2 expression to inhibit ferritinophagy	PD	[117]
Curcumol	By targeting YAP/NCOA4 to inhibit ferritinophagy	NAFLD	[118]
Schisandrin B, Oroxylin A	By regulating NCOA4-mediated ferritinophagy to promote senescence in HSCs	hepatic fibrosis	[119, 120]
Corilagin	By suppressing NCOA4-mediated ferritinophagy to regulate iron homeostasis and inhibit ferroptosis	IIRI	[123]
Esculetin	By activating NCOA4/LC3II/FTH1 signaling pathway to induce ferritinophagy and promote ferroptosis in hepatocellular carcinoma cells	Liver cancer	[124]
Ginkgolide B	By disrupting NCOA4-FTH1 interaction to inhibit ferritinophagy and alleviate CIRI	CIRI	[125]

*ART* artesunate, *DEX* dexmedetomidine, *FTH1* ferritin heavy chain 1, *NCOA4* nuclear receptor co-activator 4, *CICI* Chemotherapy-induced cognitive impairment, *CsA* Cyclosporin A, *NBP* Dl-3-n-butylphthalide, *NRF2* nuclear factor E2 related-factor 2, *PD* Parkinson’s disease, *NAFLD* non-alcoholic fatty liver disease, *HSCs* hepatic stellate cells, *IIRI* intestinal ischemia/reperfusion injury, *CIRI* Cerebral ischemia–reperfusion injury

### Summary and Prospect

The pathogenesis of CIRI is complex, and no specific drug has been developed yet. Preventing or alleviating brain damage caused by cerebral ischemia/reperfusion is a major challenge in the clinical treatment of CIRI. In recent years, the role of ferritinophagy in CIRI has received widespread attention, and NCOA4-mediated ferritinophagy may be involved in ferroptosis following CIRI by regulating iron metabolism. Therefore, an in-depth study of the specific regulatory mechanisms and effective targets of ferritinophagy in CIRI is relevant in exploring potential treatment modalities. In addition, the upstream signaling pathways that regulate ferritinophagy remain unclear, and the interactions between ferritinophagy and ferroptosis, as well as the specific mechanisms by which ferritinophagy induces ferroptosis are not fully elucidated. During the course of CIRI, different pathologic changes occur over time; therefore, the changes in pathologic processes of ferroptosis and ferritinophagy over time in CIRI should be further studied. In summary, the mechanisms underlying ferritinophagy and ferroptosis in CIRI require further research to identify effective therapeutic measures.

## Data Availability

No datasets were generated or analysed during the current study.

## Abbreviations

CIRI: Cerebral ischemia–reperfusion injury
NCOA4: Nuclear receptor coactivator 4
ROS: Reactive oxygen species
STEAP3: Six-transmembrane epithelial antigens of prostate 3
DMT1: Divalent metal transporter 1
FPN1: Membrane iron transporter 1
PUFAs: Polyunsaturated fatty acids
GPX4: Glutathione peroxidase 4
GSH: Glutathione
SLC7A11: Solute carrier family 7 member 11
CoQ10: Coenzyme Q10
FTH1: Ferritin heavy chain 1
FTL: Ferritin light
LC3B: Light chain 3 beta
HERC2: E3 ubiquitin protein ligase 2
HIF: Hypoxia-inducible factor
NRF2: Nuclear factor E2-related factor 2
MCAO/R: Middle cerebral artery occlusion/reperfusion
HO-1: Heme oxygenase-1
TfR1: Transferrin receptor 1
CIR: Cerebral ischemia–reperfusion
USP14: Ubiquitin-specific peptidase 14
cGAS: Cyclic guanylate adenylate synthetase
STING: Stimulator of interferon genes
OGD/R: Oxygen–glucose deprivation/reperfusion
